# Hyperbaric oxygen improves depression‐like behaviors in chronic stress model mice by remodeling gut microbiota and regulating host metabolism

**DOI:** 10.1111/cns.13999

**Published:** 2022-10-19

**Authors:** Bohan Zhang, Wenwen Dong, Zhixin Ma, Shuxian Duan, Ruina Han, Zhou Lv, Xinru Liu, Yanfei Mao

**Affiliations:** ^1^ Department of Anesthesiology and Surgical Intensive Care Unit, Xinhua Hospital Shanghai Jiaotong University School of Medicine Shanghai China; ^2^ Translational Medical Institute Shanghai University Shanghai China

**Keywords:** chronic stress, gut microbiota, hyperbaric oxygen, metabolism

## Abstract

**Aims:**

There is growing evidence that the gut microbiota plays a significant part in the pathophysiology of chronic stress. The dysbiosis of the gut microbiota closely relates to dysregulation of microbiota–host cometabolism. Composition changes in the gut microbiota related to perturbations in metabolic profiles are vital risk factors for disease development. Hyperbaric oxygen therapy is commonly applied as an alternative or primary therapy for various diseases. Therefore, a metabolic and gut bacteria perspective is essential to uncover possible mechanisms of chronic stress and the therapeutic effect of hyperbaric oxygenation. We determined that there were significantly disturbed metabolites and disordered gut microbiota between control and chronic stress group. The study aims to offer further information on the interactions between host metabolism, gut microbiota, and chronic stress.

**Methods:**

At present, chronic unpredictable mild stress is considered the most widespread method of modeling chronic stress in animals, so we used a chronic unpredictable mild stress mouse model to characterize changes in the metabolome and microbiome of depressed mice by combining 16S rRNA gene sequencing and UHPLC–MS/MS‐based metabolomics. Pearson's correlation‐based clustering analysis was performed with above metabolomics and fecal microbiome data to determine gut microbiota‐associated metabolites.

**Results:**

We found that 18 metabolites showed a significant correlation with *campylobacterota. Campylobacterota* associated metabolites were significantly enriched mainly in the d‐glutamate and d‐glutamine metabolism. Hyperoxia treatment may improve depression‐like behaviors in chronic stress model mice through regulating the disrupted metabolites.

**Conclusions:**

Hyperbaric oxygen improves depression‐like behaviors in chronic stress model mice by remodeling *Campylobacterota* associated metabolites.

## Graphical abstract

*Campylobacterota* was the most abundant microbiota enriched in chronic stress mice based on 16S rRNA gene sequencing. *Campylobacterota‐*associated metabolites were significantly enriched mainly in the d‐glutamate and d‐glutamine metabolism through UHPLC‐MS/MS‐based metabolomics. Hyperbaric oxygen improves depression‐like behaviors in chronic stress model mice by mainly regulating gut flora‐associated metabolites.

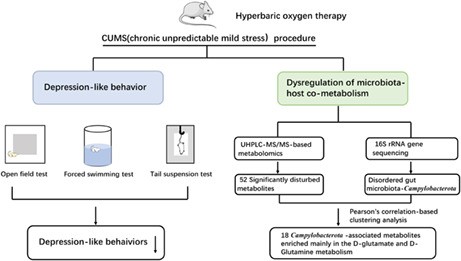

## 1 INTRODUCTION

Chronic stress refers to the body being exposed to stimulation continuously or intermittently for a long time.^1^ It is reported that the incidence of chronic stress in the United States is about 50%, of which 25% is high‐intensity stress.^2^ Chronic stress has been confirmed to promote various diseases, seriously affecting people's lives and work quality.^3^ Long‐term exposure to stressful stimuli can lead to systemic dysfunction, especially central nervous system, neuroendocrine system and immune system dysfunction.^4^ Previous studies have reported that chronic stress is one of the causes of many mental disorders, including depression and Alzheimer's disease.^5^, ^6^, ^7^ It is also a moderate risk factor for cardiovascular and cerebrovascular diseases.^8^ Therefore, there is an urgent need for new insights into the underlying pathophysiology of chronic stress to develop more effective therapeutic agents or interventions.

The mechanisms underlying the onset and development of chronic stress are now considered multifactorial, determined by multimodal changes in the gastrointestinal, endocrine, immune, central nervous system and metabolic systems.^9^ The pathological histological hallmarks of chronic stress are alterations in the hypothalamic–pituitary–adrenal (HPA) axis^10^ and the hyperactivity of sympathetic adrenal‐medullary (SAM) axes. An increasing amount of studies describe a two‐way communication between the gut flora and the host central nervous system.^9^, ^10^, ^11^ This biochemical signaling pathway, known as the brain–gut axis, can influence cognitive function and mood by regulating the nervous system, metabolism, hormones, and immunity. The gut microbiota is a key regulator of the brain–gut axis,^12^ and there is strong evidence of a connection between the gut microbiota and the pathophysiology of chronic stress through mechanisms involving vagal activation,^13^ inflammatory responses,^14^, ^15^ immunity^16^, ^17^ and gut barrier health.^18^

Dysbiosis of the gut microbiota often closely links with dysregulation of microbiota–host cometabolism.^19^ What is more, perturbations in metabolic profiles related to transformations in the gut microbiota composition are considered significant for disease development.^20^ And, in chronic stress, it has been shown that its fecal metabolic phenotype specifically changed when the gut microbiota changed.^21^ Therefore, a metabolic and gut bacteria perspective is essential to uncover possible mechanisms of chronic stress.

Hyperbaric oxygen therapy (HBOT) is a treatment procedure under a certain pressure involving 100% O_2_ breathing for a certain time. Hyperbaric oxygen therapy is gradually adapted as a basic therapy for different diseases.^22^ Moreover, Hyperbaric oxygen therapy (HBOT) gradually acts as one of the common clinical intervention methods, of which mechanism remains unclear.^23^ Therefore, in our research, we also tend to explain the therapeutic mechanism of hyperbaric oxygen from a perspective involving metabolic and gut bacteria.

Currently, chronic unpredictable mild stress (CUMS) is considered the most widespread method of modeling chronic stress in animals.^24^ It was used to study the effects of chronic stress on the occurrence, development, and aggravation of colonic inflammation, gastric precancerous lesions, depression, and other systemic diseases.^25^, ^26^, ^27^ Therefore, we used a chronic unpredictable mild stress model for our work. We used a chronic unpredictable mild stress‐induced depression mouse model to characterize changes in the microbiome and metabolome of chronic stress mice by combining 16S rRNA gene sequencing and UHPLC–MS/MS‐based metabolomics. The study aims to offer more key information about the interactions between gut microbiota host metabolism and chronic stress.

## 2 MATERIALS AND METHODS

### 2.1 Materials and methods

#### 2.1.1 Animal treatment

We purchased 28 male BALb/c mice (4 weeks of age, weighted 20 ± 2 g) from Shanghai Ji hui Laboratory Animal Care Co., Ltd. (Shanghai, China). The animals lived in the Shanghai Xinhua Hospital animal laboratory under a specific pathogen‐free environment (3 per cage). The humidity was 40%–60%, and the temperature was kept at 24 ± 2°C. The number of experimental groups is determined as four groups, the animals were weighed and then numbered in order of weight. After that, the animals were randomly assigned to the experimental groups and the control group (*n* = 7) with a randomization tool. No criteria were set about including and excluding animals. All the groups and animals were included in the analysis.

#### 2.1.2 Ethical conduct of research

All the experiments were carried out following the rules approved by the Shanghai Xinhua Hospital Ethics Committee. The study was approved by Shanghai Xinhua Hospital Ethics Committee (no. XHEC‐NSFC‐2020‐049, Feb 26th, 2020) for studies involving animals. In terms of the design of experimental animals involved in this study, the researchers strictly followed the principles “Replacement, Reduction, and Refinement” for the welfare of experimental animal and took effective care measures to protect the welfare of experimental animals and avoid unnecessary harm. The researchers have the ability and qualification to undertake the project in terms of clinical research experience and condition. The approval letter was attached as Supporting Information.

#### 2.1.3 Chronic unpredictable mild stress (CUMS) procedure

After adaptation for 1 week, mice were randomized into 4 groups (*n* = 7), including the control group (group B), CUMS model group (group D), hyperbaric oxygen treatment group (group A), and CUMS model combined with hyperbaric oxygen treatment group (group C). Mice in group C and D were housed in a different room from the other two groups, and the CUMS procedure was carried out referring to previous research.^28^, ^29^ For the CUMS mice, we used a method of stress intervention in mice at a different time, with a total of 7 kinds of random sort of stress methods, including tilted cage, damp bedding, confinement in the tube for 2 h, 5 min of 45°C oven, swimming for 5 min at 4°C, cage shaking for 10 min, exposure to empty bottles, deprivation of food and water.^29^ The procedure of CUMS is listed in Table 1.

**TABLE 1:** Chronic unpredictable mild stress (CUMS) procedure

Week	Monday	Tuesday	Wednesday	Thursday	Friday	Saturday	Sunday
First week	8:00 am–10:00 pm tilted cage	15:30 pm–17:30 pm confinement in tube for 2 h	7:00 pm–next day damp bedding	10:00 am–12:00 am cage shaking for 10 min	9:00 am–11:00 am swimming at 4°C for 5 min	9:00 am–11:00 am 45°C oven for 5 min	7:00 pm–next day space reduction
Second week	15:30 pm–17:30 pm confinement in tube for 2 h	8:00 am–10:00 pm tilted cage	10:00 am–12:00 am cage shaking for 10 min	7:00 pm–next day damp bedding	9:00 am–11:00 am 45°C oven for 5 min	9:00 am–11:00 am swimming at 4°C for 5 min	2:00 pm −2:00 am next day food and water deprivation
Third week	7:00 pm–next day space reduction	7:00 pm–next day damp bedding	9:00 am–11:00 am swimming at 4°C for 5 min	8:00 am–10:00 pm tilted cage	9:00 am–11:00 am exposure to empty bottles	15:30 pm–17:30 pm confinement in tube for 2 h	2:00 pm −2:00 am next day food and water deprivation
Fourth week	9:00 am–11:00 am 45°C oven for 5 min	9:00 am–11:00 am exposure to empty bottles	15:30 pm–17:30 pm confinement in tube for 2 h	7:00 pm–next day space reduction	10:00 am–12:00 am cage shaking for 10 min	2:00 pm −2:00 am next day food and water deprivation	8:00 am–10:00 pm tilted cage
Fifth week	15:30 pm–17:30 pm confinement in tube for 2 h	9:00 am–11:00 am swimming at 4°C for 5 min	7:00 pm–next day space reduction	9:00 am–11:00 am exposure to empty bottles	2:00 pm −2:00 am next day food and water deprivation	7:00 pm–next day damp bedding	9:00 am–11:00 am 45°C oven for 5 min

#### 2.1.4 Hyperbaric oxygen (HBO) treatment

The Control group and the CUMS model group were normally fed without stimuli or therapies. The other two groups of mice received hyperbaric oxygen (HBO) treatment. Both HBO group and combined group took hyperbaric oxygen: Sodium lime was put at the bottom of the small animal HBO chamber to absorb CO_2_, pure oxygen was consumed to wash the chamber and then was used to lift the inside pressure to 0.2 MPa for 60 min (during the span pure oxygen was used to ventilate the chamber for 10 min), 5 min was taken to reduce the pressure to normal.^22^, ^30^ The treatment remained for 21 days.^31^ When the mice were treated with HBO, the model group was put into a box which was another chamber, but the chamber gate was open, and no pure oxygen was supplied.

#### 2.1.5 Behavior test

We conducted the test in dim light during the daylight phase (from 9 a.m. to 3 p.m.), and the test included tail suspension test (TST), spontaneous activities, and forced swimming test (FST). The tests were performed as reported in previous articles.^28^, ^32^, ^33^ The condition and behavior of mice were all tracked and analyzed by Jiliang software (Shanghai Jiliang Software Technology Company, Shanghai, China). We performed the behavior test to assess whether the CUMS model succeeded and the effect of hyperbaric oxygen treatment. The behavioral analysis was finished by two researchers. One researcher conducted behavioral test and the other record the data, the researcher was blind regarding the examined group.

#### 2.1.6 sample collection

After the behavior test, mice were anesthetized with 5% isoflurane in oxygen in a plexiglass cage. After anesthesia, mice were sacrificed. The blood were extracted from heart, and fecal tissue samples were extracted from the intestine without contamination by other bacteria. Serum obtained after the left standing of blood and the fecal tissue were both stored at −80°C.

### 2.2 Fecal genome DNA extraction

CTAB/SDS was used to extract the total genomic DNA of feces. The concentration and purity of DNA were monitored on 1% agarose gel. Based on the concentration, we used sterile water to dilute DNA to 1 ng/μl.

### 2.3 Amplicon generation

We used a specific primer with the barcode (e.g., 16S V4: 515F‐806R, 18S V4: 528F‐706R, 18S V9: 1380F‐1510R, et al.) to amplify 16S rRNA/18SrRNA/ITS genes of distinct regions (16S V4/16S V3/16S V3‐V4/16S V4‐V5, 18S V4/18S V9, ITS1/ITS2, Arc V4). The PCR reactions were carried out using 15 μl of Phusion® High‐Fidelity PCR Master Mix (New England Biolabs). There were about 0.2 μM of forward and reverse primers and 10 ng template DNA in the reaction system, and the reaction was conducted under a predefined program. The thermal cycle initially denaturation at 98°C for 60 s, denaturation at 98°C for 10 s, annealing at 50°C for 30 s, and extension at 72°C for 30 s. In the end, keep the temperature at 72°C for 5 min.

### 2.4 Quantification and qualification PCR products

We mixed PCR production with an equal amount of 1× loading buffer, including SYB green. Electrophoresis detection was conducted on a 2% agarose gel. Then, we obtained the mixture of PCR products in an equal density ratio. Moreover, to purify PCR products, we used Qiagen Gel Extraction Kit (Qiagen, Germany).

### 2.5 Library preparation and sequencing

TruSeq® DNA PCR‐Free Sample Preparation Kit (Illumina, USA) was applied to generate sequencing libraries according to the instructions. Moreover, index codes were added. To assess the quality of the library, the Agilent Bioanalyzer 2100 system and the Thermo Scientific Qubit@ 2.0 Fluorometer were applied. After sequencing the library on an Illumina NovaSeq platform, 250 bp paired‐end reads were gained at last.

### 2.6 Analysis of sequencing data

Firstly, paired‐end reads were assigned to each sample based on their barcode after being generated. Then FLASH was applied to merge reads (Fast Length Adjustment of short reads, V1.2.7, http://ccb.jhu.edu/software/FLASH/)^34^ software. High‐quality clean tags^35^ filtering of the raw tags were conducted to acquire clean tags with Qiime (V1.9.1, http://qiime.org/scripts/split_libraries_fastq.html).^36^ We compared the tags with the reference database (Silva database, https://www.arb‐silva.de/) using UCHIME algorithm (UCHIME Algorithm, http://www.drive5.com/usearch/manual/uchime_algo.html).^37^ Then, chimera sequences were detected and removed.^38^ Finally, the Effective Tags were obtained. Uparse (Uparse v7.0.1001, http://drive5.com/uparse/)^39^ software was applied to perform sequences analysis. Sequences with a similarity of ≥97% are classified as the same operational taxonomic unit (OTU). To annotate taxonomic information, we used the Silva Database (http://www.arb‐silva.de/)^40^ for each representative sequence based on the Mothur algorithm. Then, the phylogenetic relationship of different OTUs was studied. What is more, the dominant species difference in different samples (groups) was also considered. In order to conduct multiple sequence alignment, we used the MUSCLE software (Version 3.8.31, http://www.drive5.com/muscle/).^41^ In order to normalize the abundance information of OTUs, we performed the corresponding sequence number standard for the sample with the least sequences. Then, we classified typical sequences for every OUT. Moreover, *α* diversity and *β* diversity were performed to identify the species diversity complexity for each sample. The diversity in samples was assessed by calculations analysis with principal coordinate analysis (PCoA). Then, we compared bacterial abundance and diversity. Wilcoxon rank‐sum test and Welch's *t*‐test were classical study methods. At the genus level, Nonparametric Wilcoxon test (*p* < 0.05, *q* < 0.1) was applied to construct heat maps. Finally, linear discriminant analysis (LDA) coupled with effect size (LEfSe) was applied to evaluate the differentially abundant taxon.

### 2.7 Sample preparation for metabolomics analysis

The frozen plasma samples were thawed at room temperature. After gently mixing, a 100 μl aliquot of plasma was added to 300 μl of methanol, containing 5 μg/ml chlorophenylalanine as internal standard, vortexed for 30 s. After centrifugation (12,000 rpm, 4°C, 10 min), the deproteinized supernatant was transferred to 1.5 ml sample vials with inserts for LC–MS analysis. Pooled quality control (QC) samples were prepared by mixing equal amounts (10 μl) of tissue and plasma samples, which was a part of the system conditioning and QC process.

### 2.8 UPLC–MS/MS data acquisition

Metabolites were separated on an Ultimate 3000 UHPLC system (Thermo Scientific). We used a ZIC‐pHILIC column (2.1 mm i.d. × 100 mm, 5 μm, Merck) at 30°C to separate the compound. Mobile phase A was water with 20 mM ammonium carbonate and 0.2% ammonium hydroxide solution, and mobile phase B was acetonitrile. The flow rate was 0.2 ml/min. The samples were suspended in 100 μl of acetonitrile: water (1:1, v/v) solution. The injection volume is 1 μl. Ion detection was performed using Thermo Scientific Q‐Exactive MS with an electrospray (ESI) source simultaneously operating in fast negative/positive ion switching mode. The acquisition setting was applied in full scan mode with the following parameters to collect metabolomics data. The parameter is listed in Table 2.

**TABLE 2:** MS acquisition parameters in full scan mode to collect metabolomics data

HESI source	Q exactive
Sheath gas = 40	Pos.Ion (70–1050 amu)
	Neg.Ion (70–1050 amu)
Aux gas = 10	MS resolution: *R* = 70,000, (FWHM at *m/z* 200)
Spray voltage: +3.5/−4.0 KV	MS isolation width = 1 Da
RF‐lens: 50	AGC target 3E6 MS, 200 ms max
Cap. temp: 300°C	
Heater temp: 350°C	

### 2.9 Metabolomics data analysis based on UHPLC–MS/MS

We used open‐source software MZmine v2.32^42^ to process the raw data of metabolomics generated by UPLC‐MS/MS. Then, we performed targeted feature detection (feature list files consisted of reference compounds in our self‐build metabolomics library), deisotoping (*m/z* tolerance was 0.001; RT tolerance was 6 s), alignment (join aligner *m/z* tolerance was 1 ppm; RT tolerance was 30 s; weight for *m/z* was 15 and for RT was 10), gap filling (intensity, *m/z*, and RT tolerance were 1%, 0.001 *m/z*, and 30 s, respectively), and dereplication. For further statistical analysis, a few non‐peak‐shape features were removed by visual inspection and exported both data sets from MZmine as csv files. The files included retention time, *m/z*, area of each peak, and the identification number. We used SIMCA‐P version 14.0 (Umetrics AB, Umea, Sweden) and then performed principal component analysis (PCA), orthogonal projection to latent structure–discriminant analysis (PLS‐DA), and multivariate statistical methods. Student *t*‐test and fold change were applied to measure the significance of each metabolite. Spearman rank correlation analysis, pathway analysis and heat maps were performed with MetaboAnalyst5.0 (http://www.metaboanalyst.ca/).

### 2.10 Statistical analysis

All data were analyzed with the statistical program SPSS 25.0 (Chicago, IL, USA). Data are expressed as means ± SEM. Statistical comparisons between experimental groups and control group were performed using two‐sample *t*‐test (*T*–*T* test) with an additional Bonferroni post hoc test. Multiple groups were compared using one‐way ANOVA test followed by Bonferroni correction. Statistical significance was defined as *p*‐value <0.05.

## 3 RESULTS

### 3.1 Depression‐like behavior in the CUMS mice

**FIGURE 1:**
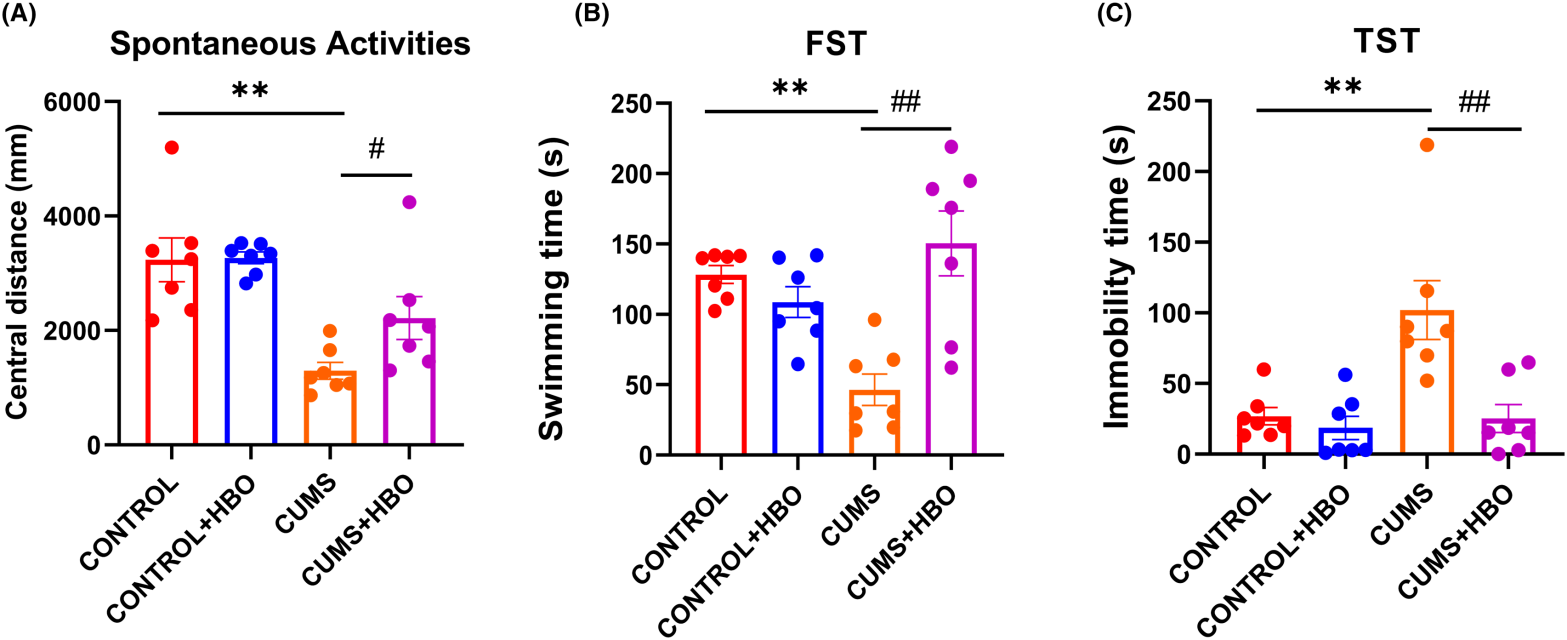
Depression‐like behavior in four groups. (A) Central distance of spontaneous activities in four groups. (B) Swimming time in FST in four groups. (C) Immobility time in TST in four groups. The results are expressed as mean ± SEM, *n* = 7 in group A–D. **p < 0.01 compared to control group, ^#^ *p* < 0.05, ^##^ *p* < 0.01 compared with CUMS model group.

We analyzed the depression‐like behavior in different groups after 35 days of CUMS modeling. In spontaneous activities, central distance (Figure 1A) was analyzed to confirm the success of CUMS model. The values were 3226 ± 380.8 mm in the control group, 3140 ± 115.6 mm in the HBO group, 1296 ± 148.5 mm in CUMS group, and 2217 ± 374.2 mm in CUMS combined HBO group. In forced swimming test (FST), swimming time (Figure 1B) was recorded to analyze the success of CUMS model. The values were 35.99 ± 7.124 s in the control group, 49.46 ± 3.694 s in the HBO group, 26.81 ± 6.097 s in CUMS group, and 46.93 ± 11.01 s in CUMS combined HBO group. In tail suspension test (TST), swimming time (Figure 1C) was analyzed. The values were 23.27 ± 9.315 s in the control group, 9.970 ± 3.717 s in the HBO group, 13.16 ± 5.323 s in CUMS group, and 51.19 ± 13.90 s in CUMS combined HBO group. According to the results, compared with the control group, CUMS group performed shorter central distance (*p* < 0.001) in spontaneous activities, less swimming time (*p* < 0.0001) in FST, and more immobility time (*p* < 0.01) in TST. The figure illustrated that the CUMS model was in a condition of depression. However, compared with the CUMS group, the CUMS mice after HBO treatment showed longer central distance (*p* < 0.05) in spontaneous activities, more swimming time (*p* < 0.01) in FST, and less immobility time (*p* < 0.01) in TST. The results showed the protective effect of hyperbaric oxygen in CUMS‐induced depression behavior.

### 3.2 Disordered gut microbiota between control mice and chronic stress mice

According to our present microbiome investigation, we gained 2,346,691 high‐quality 16S rRNA reads in total. Each sample had a median read count of 98,173 (range, 55,982 to 109,181). Then, we obtained 2710 OTUs after the taxonomic assignment. The species accumulation curve (shown in Figure S1A) and the rarefaction curve (shown in Figure S1B) of all samples explained that sufficient sampling work was carried out.

**FIGURE 2:**
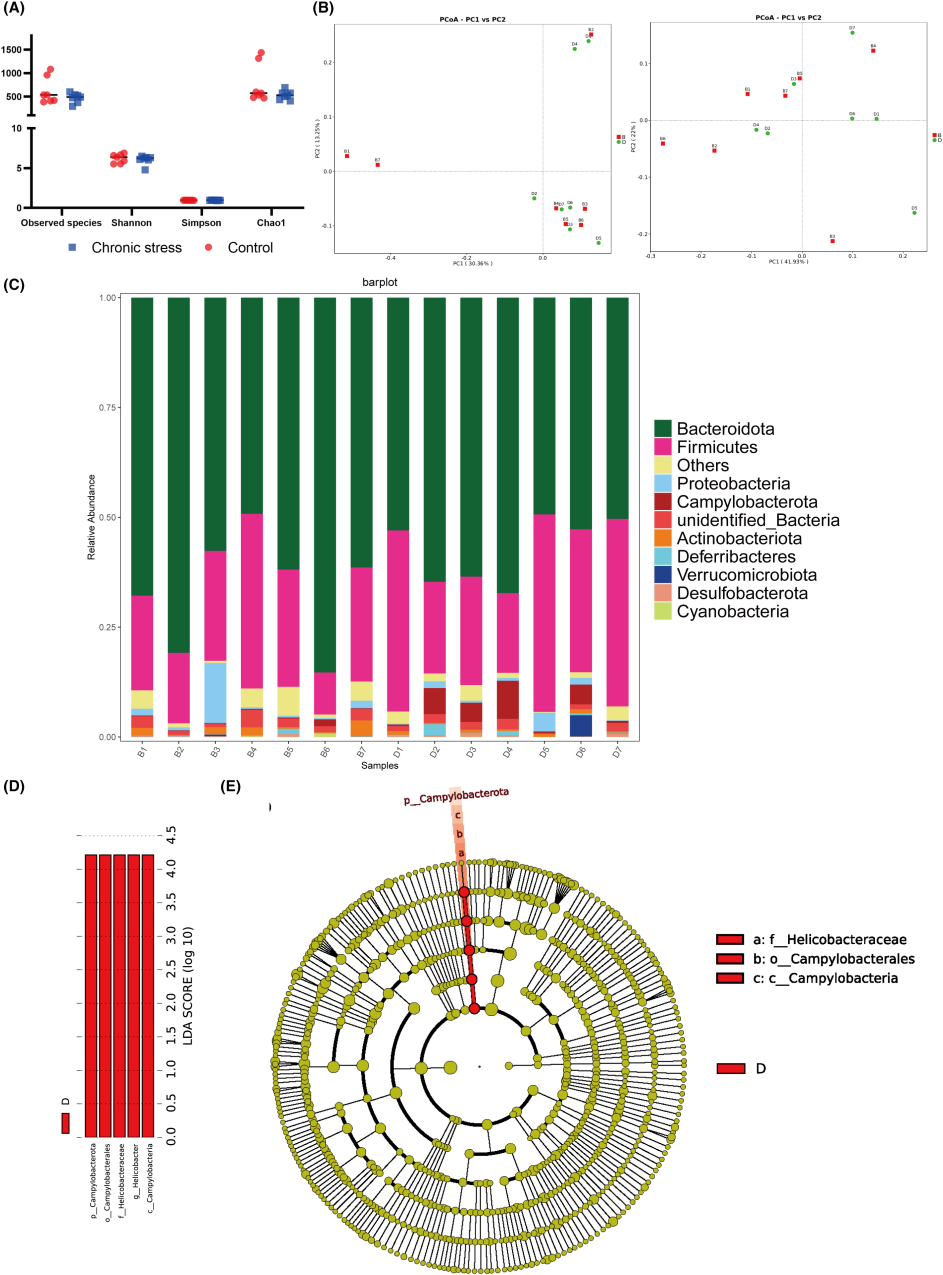
Analysis of gut structure and microbiome diversity. (A) Differences in species diversity between the stress and control groups were estimated by the observed species, Shannon, Simpson, and Chao1 indices. (B) PCoA plot based on the relative abundance of OTUs (97% similarity level) demonstrating the bacterial structural clustering. (i) Unweighted UniFrac PCoA plots; (ii) weighted UniFrac PCoA plots. Chronic stress group (red dots); control group (blue dots). Dots represent individual samples. (C) Proportion of component in bacterial phylum of each group; B, control group; D, chronic stress group. (D) linear discriminant analysis (LDA) with effect size (LEfSe) and the differences in abundance between the control and chronic stress groups (E) Cladogram illustrating the phylogenetic distribution of microbiota related to the chronic stress or control groups.

α diversity and β diversity were valid methods to explore the differences in bacterial diversity. Therefore, we sorted the sequences. The observed species showed no statistically significant differences (618.57 ± 282.99 vs. 470.86 ± 104.02, *p* = 0.219), Shannon (6.21 ± 0.56 vs. 6.04 ± 0.57, *p* = 0.58), Simpson index (0.97 ± 0.01 vs. 0.96 ± 0.03, *p* = 0.607), and Chao1 indexes (772.1 ± 416.57 vs. 531.16 ± 95.27, *p* = 0.162) between control mice and stress mice (Figure 2A). No obvious difference between the two groups based on the first three PCoA (Figure 2B), according to the unweighted PCoA plots and the weighted PCoA plots. Moreover, microbial taxon assignment was conducted in both groups to evaluate the relative proportions of dominant taxa at the phylum level. In each group, we identified the top ten phyla (Figure 2C). *Bacteroidetes* was the main phylum, taking a total of 66.3% and 57.3%, respectively, of the OTUs in the control and stressed groups. Moreover, *Campylobacterota* (0.36% vs. 3.52%, *p* = 0.026) was enriched in the depressed group. We made a cladogram using LEfSe to search out the typical bacteria related to stress‐induced depression. At last, 5 discriminatory OTUs were identified as key discriminants. In the depressed group, the most abundant microbiota was *Campylobacterota* (LDA scores (log10) > 4). (Figure 2D).

### 3.3 Significantly disturbed metabolites between control and chronic stress group

**FIGURE 3:**
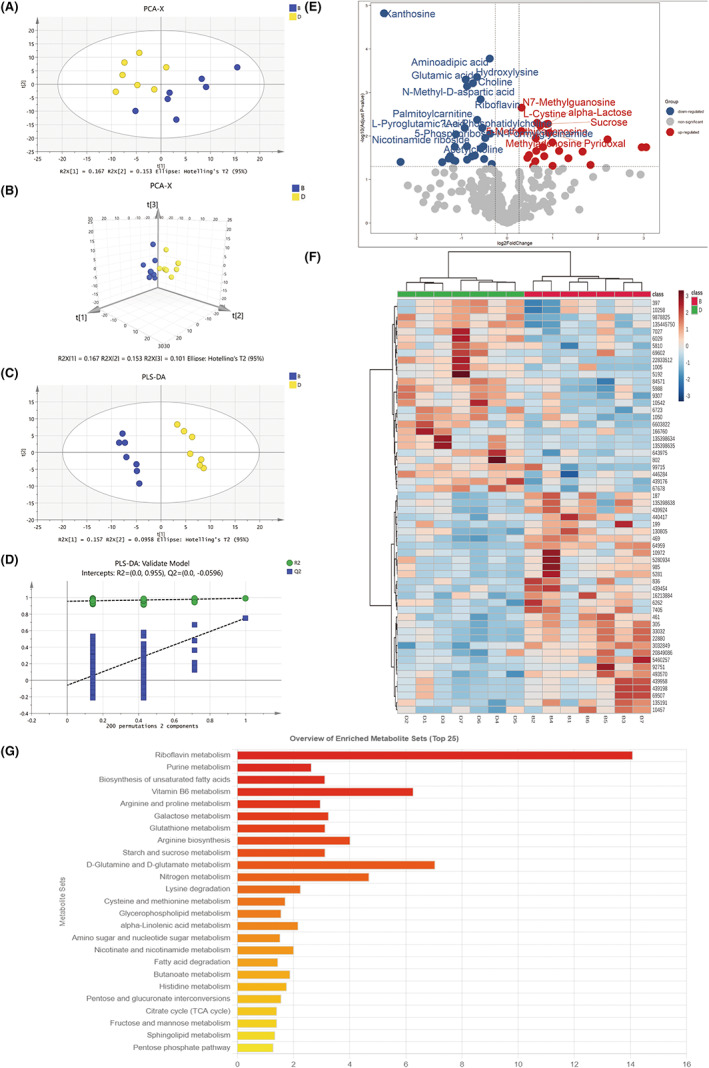
Comparative metabolomics analysis of depression and control group. (A) PCA 2D score plot (B) PCA 3D score plot. (C) PLS‐DA 2D score plot (D) Validation plot of PLS‐DA model using a 200 times permutation test. (E) Volcano Plot comparing depression and control group. (F) Heatmap of 52 different metabolites. (G) KEGG pathway enrichment analysis of 52 differential metabolites. B, control group; D, chronic stress group. The cutoff *p*‐value has a corresponding *p*‐value of <0.05 and a fold change cutoff value of 1.5.

Metabolomics analysis was performed between CUMS mice and control mice. We employed UPLC–MS/MS and used reference compounds and a metabolite database to identify 310 metabolites in total. Our metabolic profiles were evaluated by unsupervised and supervised statistics. The PCA score plot with the first two (Figure 3A) and three (Figure 3B) principal components showed an obvious difference between the control and model group. In the score plot of the supervised PLS‐DA model, the depressed group was separated completely from the control group (Figure 3C), the PLS‐DA model was satisfactorily validated with 200 permutation tests (*R* ^2^‐int = 0.955, *Q* ^2^‐int = −0.0596) (Figure 3D). We performed supervised OPLS‐DA to find the potential biomarkers contributing to chronic stress‐induced depression. The volcano plot analysis for the control and depressed groups showed 52 significant variables (Figure 3E). 52 metabolites with a VIP value of >1.0, *p*‐value of <0.05, and fold change of >1.2 for each comparison were screened out as significantly changed metabolites caused by chronic stress. The expression heat map of the 52 metabolites is shown in Figure 3F, and they are significantly enriched mainly in the Riboflavin metabolism and d‐glutamate and d‐glutamine metabolism (Figure 3G).

### 3.4 Correlation between the differential metabolites and bacteria taxa

**FIGURE 4:**
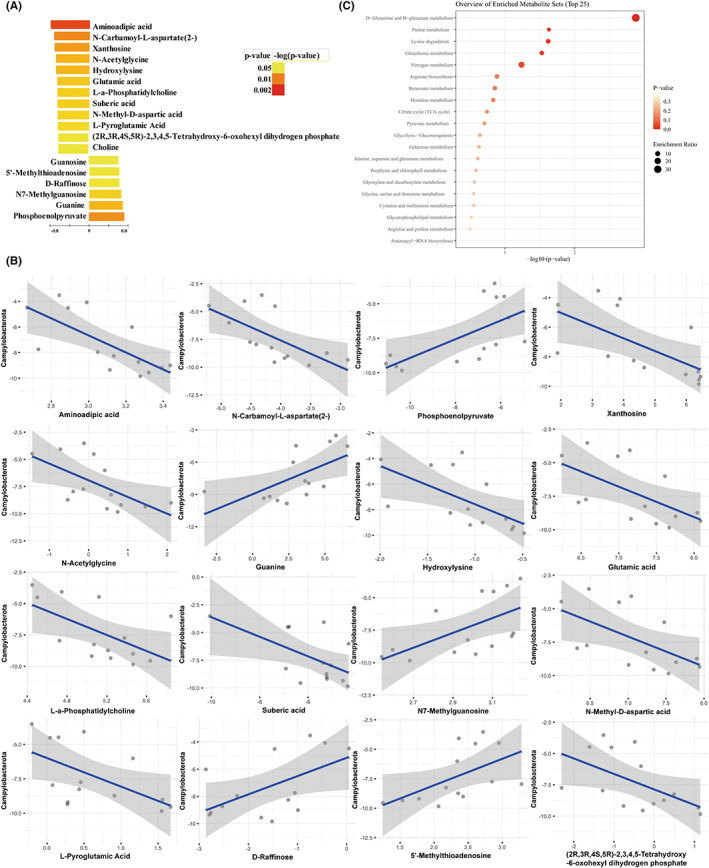
Correlations between the differential metabolites and bacteria taxa. (A) Heatmap of correlations between differential metabolites and taxa. (B) Scatter plots display the relationship between differential metabolites and *campylobacterota*. (C) KEGG pathway enrichment analysis of 18 *campylobacter*‐associated metabolites.

To search out microbe‐associated metabolites in depressed mice, Pearson's correlation‐based clustering analysis was performed with above metabolomics and fecal microbiome data. We found that 18 metabolites showed a significant correlation with *campylobacterota* (Figure 4A). Among them, (i) aminoadipic acid (*r* = −0.71, *p* = 0.004), N‐carbamoyl‐l‐aspartate(2‐) (*r* = −0.648, *p* = 0.012), xanthosine (*r* = −0.632, *p* = 0.015), N‐acetylglycine (*r* = −0.61, *p* = 0.021), hydroxylysine (*r* = −0.606, *p* = 0.021), glutamic acid (*r* = −0.59, *p* = 0.026), l‐α‐phosphatidylcholine (*r* = −0.582, *p* = 0.029), suberic acid (*r* = −0.581, *p* = 0.03), N‐Methyl‐d‐aspartic acid (*r* = −0.575, *p* = 0.031), l‐pyroglutamic acid (*r* = −0.567, *p* = 0.034), (2R, 3R, 4S, 5R)‐2,3,4,5,‐tetrahydroxy‐6‐oxohexyl dihydrogen phosphate (*r* = −0.544, *p* = 0.044), and choline (*r* = −0.543, *p* = 0.045) were significantly negative correlated with *campylobacterota*; (ii) guanosine (*r* = 0.537, *p* = 0.048), 5′‐methylthioadenosine (*r* = 0.545, *p* = 0.044), d‐Raffinose (*r* = 0.548, *p* = 0.042), N7‐Methylguanosine (*r* = 0.58, *p* = 0.03), guanine (*r* = 0.609, *p* = 0.021), and phosphoenolpyruvate (*r* = 0.643, *p* = 0.013) were significantly positive correlated with *campylobacterota* (Figure 4B)*. Campylobacterota* associated metabolites were significantly enriched mainly in the d‐glutamate and d‐glutamine metabolism (Figure 4C). The information of the 18 metabolites that showed a significant correlation with *campylobacterota* are listed in Table 3.

**TABLE 3:** Metabolites significantly correlating with *campylobacterota*

Metabolites	Formula	HMDB ID	log2FC	*p*‐value	Pathway
Aminoadipic acid	C6H11NO4	HMDB0000510	−0.3853	***	Lysine degradation
N‐Carbamoyl‐l‐aspartate	C5H8N2O5	HMDB0000828	−0.77899	*	Aspartate metabolism
Xanthosine	C10H12N4O6	HMDB0000299	−2.7067	***	Purine metabolism
N‐Acetylglycine	C4H7NO3	HMDB0000532	−1.1725	*	
Hydroxylysine	C6H14N2O3	HMDB0000450	−0.66641	***	
Glutamic acid	C5H9NO4	HMDB0000148	−0.90843	***	Alanine metabolism
l‐α‐Phosphatidylcholine	C40H80NO8P	HMDB0000564	0.62395	**	Phospholipid biosynthesis
Suberic acid	C8H14O4	HMDB0000893	−1.1509	*	
N‐Methyl‐d‐aspartic acid	C5H9NO4	HMDB0002393	−0.87873	***	
l‐Pyroglutamic acid	C5H7NO3	HMDB0000267	0.60175	*	Glutathione metabolism
(2R,3R,4S,5R)‐2,3,4,5‐Tetrahydroxy‐6‐oxohexyl dihydrogen phosphate	C6H13O9P	HMDB0242558	−1.1392	*	
Choline	C5H14NO	HMDB0000097	−0.75373	***	Betaine metabolism
Guanosine	C10H13N5O5	HMDB0000133	3.0545	*	Purine metabolism
5′‐Methylthioadenosine	C11H15N5O3S	HMDB0001173	0.72614	**	Methionine metabolism
d‐Raffinose	C18H32O16	HMDB0003213	1.1397	*	Galactose metabolism
N7‐methylguanosine	C11H18N5O11P2	HMDB0059613	0.31739	**	
Guanine	C5H5N5O	HMDB0000132	2.9533	*	Purine metabolism
Phosphoenolpyruvate	C3H5O6P	HMDB0000263	1.6594	*	Amino sugar metabolism

*Note*: Log2FC > 0.26 the metabolite increases significantly; Log2FC < −0.26: the metabolite decreases significantly.

* *p* < 0.05;

** *p* < 0.01;

*** *p* < 0.001.

### 3.5 Influence of hyperoxia therapy on metabolism and gut microbiota

**FIGURE 5:**
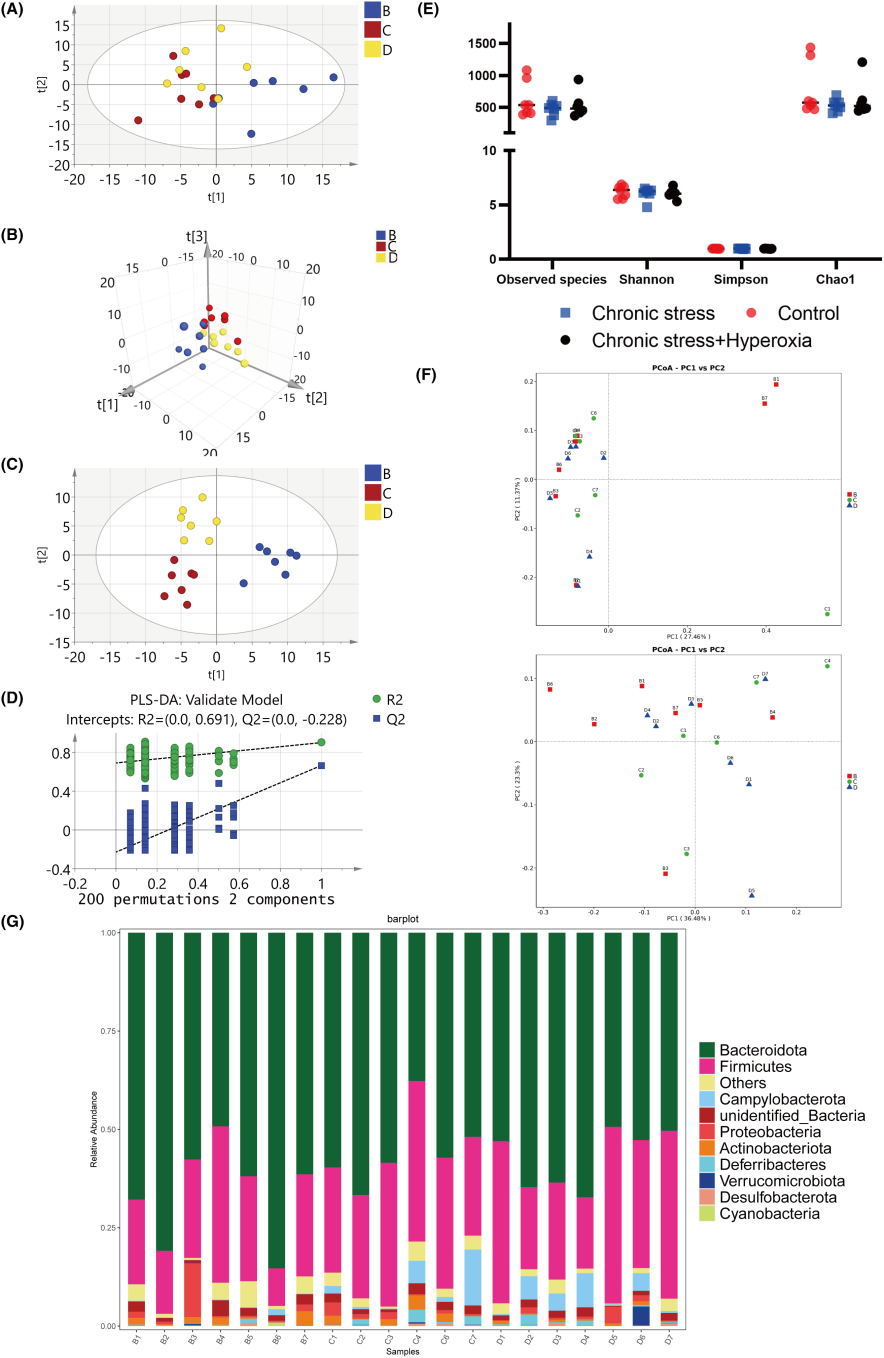
Analysis of metabolism and gut microbiota after hyperoxia treatment. (A) PCA 2D score plot. (B) PCA 3D score plot. (C) PLS‐DA 2D score plot (D) Validation plot of PLS‐DA model using a 200 times permutation test. (E) The differences in species diversity between the model group, the hyperoxia treatment group and the control group were estimated through the Shannon, Simpson, observed species, and Chao1 indexes. Differences in species diversity between the model, hyperoxia‐treated and control groups were estimated by the observed species, Shannon, Simpson, and Chao1 indices. (F) PCoA plot based on the relative abundance of OTUs (97% similarity level) illustrating bacterial structural clustering. (i) Unweighted UniFrac PCoA plots; (ii) weighted UniFrac PCoA plots. Chronic stress group (red dots); control group (blue dots); hyperoxia‐treated group (yellow dots), where dots represent individual samples. (G) Component proportion of bacterial phylum in each group; B, control group; C, hyperoxia‐treated group; D, chronic stress group.

On the 2D‐PCA score plot, the hyperoxia‐treated samples were partially clustered with depression samples (Figure 5A), while on the 3D‐PCA score plot, there was a clear distinction between the three groups (Figure 5B). We further developed a PLS‐DA model and found that the hyperoxia‐treated group showed a tendency to retrace toward the control group on the second principal component of the PLS‐DA model (Figure 5C), which indicated that hyperoxia might improve depression induced by chronic stress. We used a validation plot to visualize cluster of 200 permutated models, and the *R* ^2^‐int and *Q* ^2^‐int values were 0.683 and − 0.243, respectively (Figure 5D). At the same time, we evaluated the differences in bacterial diversity after hyperoxia treatment. We found that the three groups did not differ obviously in alpha diversity (Figure 5E), beta diversity (Figure 5F), and the relative proportions of dominant taxa at the phylum level (Figure 5G).

**FIGURE 6:**
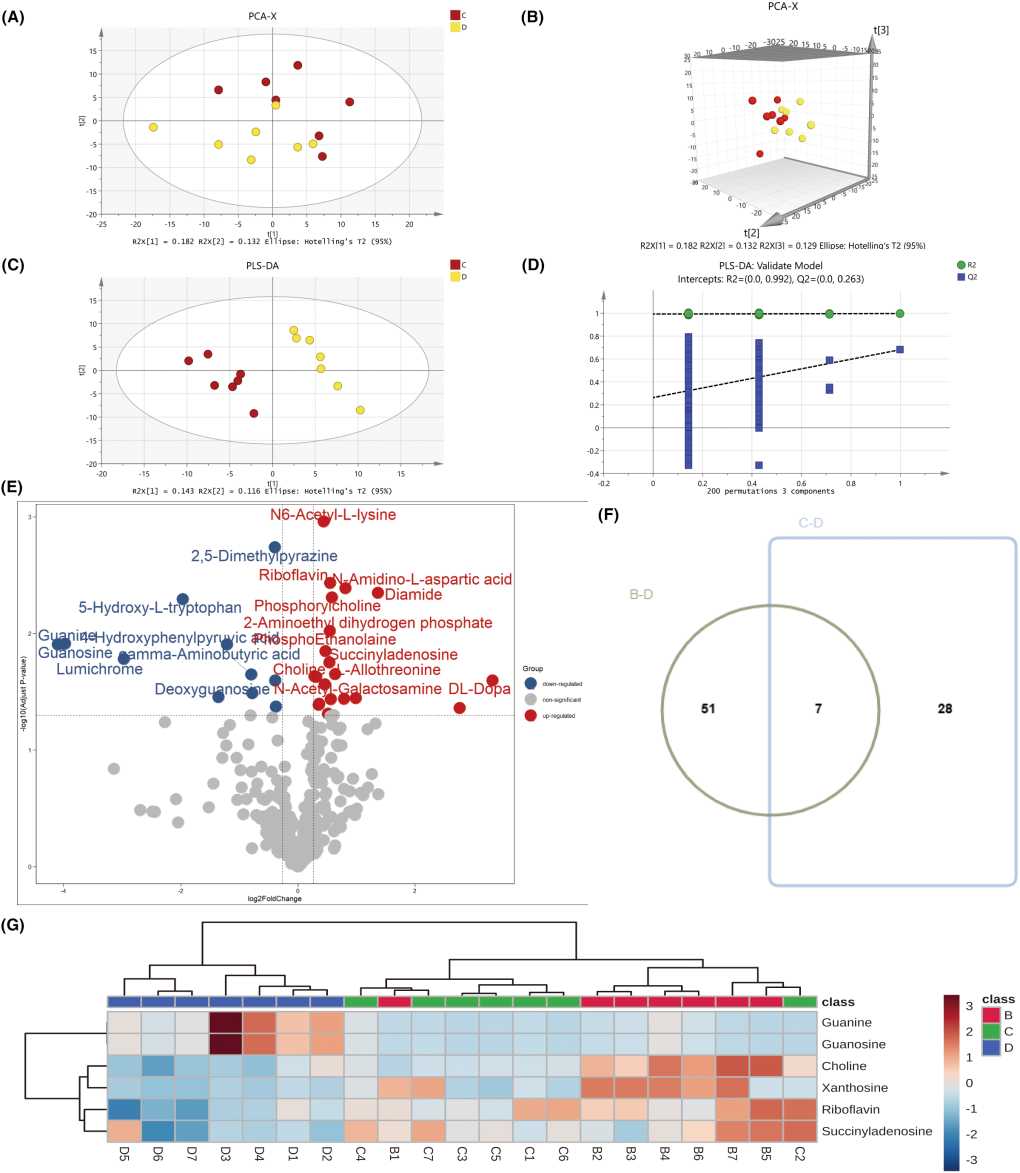
Comparative metabolomics analysis of depression and hyperoxia‐treated group. (A) PCA 2D score plot. (B) PCA 3D score plot. (C) PLS‐DA 2D score plot. (D) Validation plot of PLS‐DA model using a 200 times permutation test. (E) Volcano plot comparing depression and hyperoxia‐treated group. (F) Venn plot of differential metabolites obtained from B vs. D and C vs. D. (G) Heatmap of 8 metabolites. B, control group; C, hyperoxia‐treated group; D, chronic stress group. The cutoff *p*‐value has a corresponding *p*‐value of <0.05 and a fold change cutoff value of 1.5.

The overall metabolic profiles of hyperoxia‐treated and depressed groups showed a clear trend toward separation on both two‐(Figure 6A) and three‐dimensional PCA (Figure 6B) and PLS‐DA score plots (Figure 6C). The *R* ^2^‐int and *Q* ^2^‐int values of the permutated model were 0.683 and − 0.243, respectively (Figure 6D). A total of 30 significant variables were identified by volcano plot analysis (Figure 6E), and eight of them overlapped with depression‐related metabolites (Figure 6F). After hyperoxia treatment, the eight metabolites disrupted in the depression group were significantly back‐regulated toward the control group (Figure 6G).

### 3.6 Metabolic mechanism of the therapeutic effect of hyperoxia on depression in mice

**FIGURE 7:**
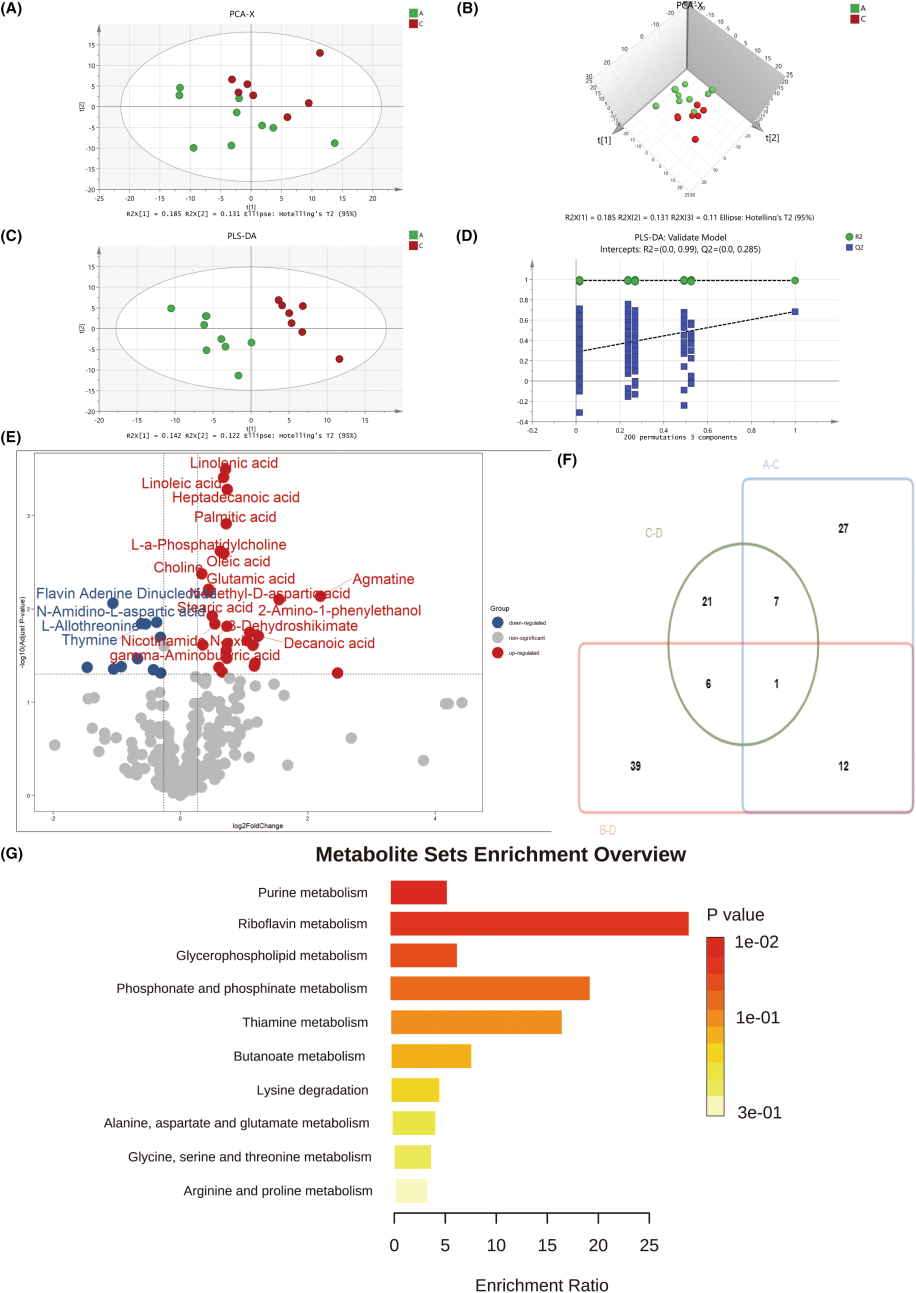
Comparative metabolomics analysis of hyperoxia‐treated group and normal mice with hyperoxia. (A) PCA 2D score plot. (B) PCA 3D score plot. (C) PLS‐DA 2D score plot. (D) Validation plot of PLS‐DA model using a 200 times permutation test. (E) Volcano plot comparing hyperoxia‐treated group and normal mice with hyperoxia. (F) Venn plot of differential metabolites obtained from B vs. D, C vs. D, and A vs. C. (G) KEGG pathway enrichment analysis of 12 differential metabolites. A, normal mice with hyperoxia; B, control group; C, hyperoxia‐treated group; D, chronic stress group. The cutoff *p*‐value has a corresponding *p*‐value of <0.05 and a fold change cutoff value of 1.5.

Treated with hyperoxia, the overall metabolic profiles of control and depressed groups showed complete separation on both two‐ (Figure 7A) and three‐dimensional PCA (Figure 7B) and PLS‐DA score plots (Figure 7C). The *R* ^2^‐int and *Q* ^2^‐int values of the permutated model were 0.99 and 0.285, respectively (Figure 7D). Analysis using volcano plots revealed significant differences in 32 metabolites (Figure 7E), and ten of them overlapped with depression‐related metabolites (B–D vs. A–C) (Figure 7F), suggesting that these ten disease‐related metabolites may respond poorly to hyperoxia treatment. Of the metabolites that changed after hyperoxia treatment in depressed mice (C–D), eight overlapped with B–D and five with A–C (Figure 7F). These 12 metabolites were enriched significantly in Glycerophospholipid and riboflavin metabolism, which may be related to the mechanism of hyperoxia treatment of stress‐induced depression (Figure 7G).

## 4 DISCUSSION

The metabolic profile in plasma was investigated in a chronic stress state in our study, and we found that riboflavin metabolism is the most significantly altered metabolic pathway in mice with chronic stress‐induced depression. Riboflavin is a water‐soluble member of the B vitamin family, and deficiencies of group B vitamins may be associated with chronic stress during the aging process.^43^ The relationship between riboflavin and chronic stress has been less studied than other B vitamins, but in an earlier study, it was found that 27% of psychiatric in‐patients had riboflavin deficiency.^44^ Riboflavin has a wide range of biological effects, improving glutamate excitotoxicity neuroinflammation, mitochondrial dysfunction and oxidative stress.^45^ These were all implicated according to current mechanisms of depression‐like behaviors relating to chronic stress. In addition, riboflavin is a key factor affecting homocysteine metabolism, pyridoxine activation, and tryptophan‐kynurenine pathway. Particularly, pyridoxal phosphate, which is the active form of pyridoxine, is a neuroprotective factor, and the accumulation of homocysteine or kynurenine in the body due to riboflavin deficiency can lead to serious neurological consequences.^45^ Thus, riboflavin has a potential neuroprotective effect, and its deficiency may be one of the key factors contributing to depression‐like behavior induced by chronic stress.

There is growing evidence of a potential association between gut flora dysbiosis and chronic stress,^46^ two independent studies in 2016 found that both mice and rats transplanted with fecal microbes from humans in depressive condition exhibited a highly inflammatory state and depression‐like behaviors.^10^, ^46^ Therefore, the present study established a mouse model of chronic stress‐induced depression to find out the association between changes in chronic stress and gut microbiota. We found that *Campylobacter* was increased at the family, order, class, and genus levels in mice with chronic stress. The relevance of *Campylobacter* to chronic stress has received little attention, with *Campylobacter* infection mainly affecting the gastrointestinal tract, musculoskeletal system, etc.^47^ However, some studies have shown that *Campylobacter* infection increases scores in anxiety, depression, somatization, and neurotic traits in patients while inducing acute inflammatory gastroenteritis.^48^, ^49^

Symbiotic bacteria in gut may experience a microbial imbalance, and the phenomenon was known as “dysbiosis”.^50^ The loss of gut microbiota diversity is associated with increased damage of intestinal mucosal and the fragility of gut. Notably, reduced production of SCFAs along with abnormal gut microbiota not only accelerated NF‐κB‐mediated inflammation but also increased intestinal permeability, leading to bacterial translocation and higher levels of IL‐6 and TNF‐α.^51^ The dysregulation of gut microbiota could cause neuropsychiatric behaviors via release of pro‐inflammatory factors.^52^ Specific inflammation‐relevant microbiota exists in central and peripheral organs and can serve as disease biomarkers.^53^ The gut microbiome, especially those associated with inflammation, may reflect dysbiosis in the brain and gut.^52^ Neuronal inflammation induced by intestinal inflammation lead to neuronal loss and finally lead to neurological diseases such as stroke, Alzheimer's disease, and central nervous system injury.^54^

Gut microbiota is well known to have an important influence on host metabolism. The dysregulation of the microbiota–host co‐metabolism is often related to dysbiosis.^55^ Therefore, we propose to explore the role of *Campylobacter* abnormalities in the pathogenesis of chronic stress in terms of host metabolism and gut bacteria relationships. We performed a Pearson correlation analysis of 52 chronic stress‐related metabolites and *Campylobacter* abundance obtained from this experiment and found that 18 metabolites, including aminoadipic acid, were significantly correlated with changes in *Campylobacter* in depressive states, and these 18 metabolites were significantly enriched in D‐glutamate metabolism and d‐glutamine. Glutamate (Glu) is one of the main excitatory neurotransmitters in the central nervous system and is synthesized in neurons primarily from glutamine (Gln), which is then released into the synaptic gap. Abnormalities in the Glu–Gln cycle play an important part in the pathophysiology of chronic stress.^56^, ^57^ Currently, the changes in Gln and Glu levels in depressed patients are not well defined,^58^ and our study found that levels of the glu pathway were significantly reduced in depressed mice and were closely associated with changes in *Campylobacter*, which could potentially be associated with the development of chronic stress by affecting glutamate metabolism.

Early studies of cerebral blood flow in patients with chronic stress have shown a significant reduction in blood flow to the left hemisphere in depressed patients.^59^ Hyperbaric oxygen rapidly increases arterial partial pressure and blood oxygen levels in the body, improving hypoxic symptoms. We found that the overall metabolic profile of depressed mice treated with hyperoxia tended to return to normal. However, abnormalities in the gut flora did not improve, suggesting that metabolic abnormalities are more sensitive to hyperoxia treatment and that hyperoxia treatment may exert its effects on chronic stress by improving Glycerophospholipid metabolism and riboflavin metabolism in mice. Further experimental verification is needed to determine the role these metabolism and gut flora played in stress‐induced depression. It is worth noting that sex differences exist in cerebral blood flow, neuroinflammation, gut microbiota, and neuropsychiatric behaviors. Previous study showed that ischemic stroke leads more women than men to death, and sex difference in microglia are an important factor in the inflammatory response after ischemic injury.^60^ There is recent evidence that different rates of cortical energy turnover and blood flow exists in men and women because men have higher density of synapses in temporal cortex than women.^61^ Sex also has an impact on age‐related cerebral perfusion trajectories in cognitively asymptomatic middle‐aged and older adults due to the difference in regional decreased cerebral perfusion between males and females.^62^ Therefore, study of females was our limitation and data from both sexes would be addressed in our future studies. Furthermore, whether we can use human serum samples and fecal samples is also important to confirm our views.

## AUTHOR CONTRIBUTIONS

Yanfei Mao and Xinru Liu conceived of the study, contributed to the formulation of overarching research goals, developed the proposal, and initiated the writing of the manuscript. Bohan Zhang and Ruina Han were responsible for data analysis and writing of the manuscript. Wenwen Dong and Zhixin Ma were responsible for clarifying the manuscript content and word usage for an English language audience. Wenwen Dong and Zhou Lv were responsible for sample extraction and LC‐MS method development. Bohan Zhang and Zhou Lv conducted behavior test and were responsible for storage of samples.

## FUNDING INFORMATION

The present study was supported by grants from the National Natural Science Foundation of China (grant no. 81772108).

## CONFLICT OF INTEREST

The authors have no other relevant affiliations or financial involvement with any organization or entity with a financial interest in or financial conflict with the subject matter or materials discussed in the manuscript apart from those disclosed. No writing assistance was utilized in the production of this manuscript.

## Supporting information


Appendix S1

Appendix S2

Appendix S3

## Data Availability

All data are contained within the manuscript. This article contains supplemental data.
