# Supplementary material for: Hyperbaric oxygen improves depression‐like behaviors in chronic stress model mice by remodeling gut microbiota and regulating host metabolism
Source: CNS Neurosci Ther. 2022 Oct 19;29(1):239–55. doi: 10.1111/cns.13999 (PMC9804075; doi:10.1111/cns.13999)
Supplement: Supplementary file 3 — Appendix S3 [file CNS-29-239-s002.docx]

**Hyperbaric oxygen improves depression-like behaviors in chronic stress model mice by remodeling gut microbiota and regulating host metabolism**

Bohan Zhang^1＃^, Shuxian Duan^2＃^, Zhixin Ma^2^, Zhou Lv^1^, Xinru Liu^2*^，Yanfei Mao^1*^

1 Department of Anesthesiology and Surgical Intensive Care Unit, Xinhua Hospital, Shanghai Jiaotong University School of Medicine, Shanghai 200092, PR China

2 Translational Medical Institute, Shanghai University, Shanghai 200444, PR China

*Correspondence to: Yanfei Mao, Department of Anesthesiology and Surgical Intensive Care Unit, Xinhua Hospital, Shanghai Jiaotong University School of Medicine, Shanghai 200092, PR China，E-mail: maoyanfei@xinhuamed.com.cn or Xinru Liu, Translational Medical Institute, Shanghai University, Shanghai 200444, PR China, E-mail: liuxinru@hotmail.co.uk

#Bohan Zhang and Shuxian Duan contributed equally to this study.

The Supplementary material contains 1 figure

**Figure S1**

**
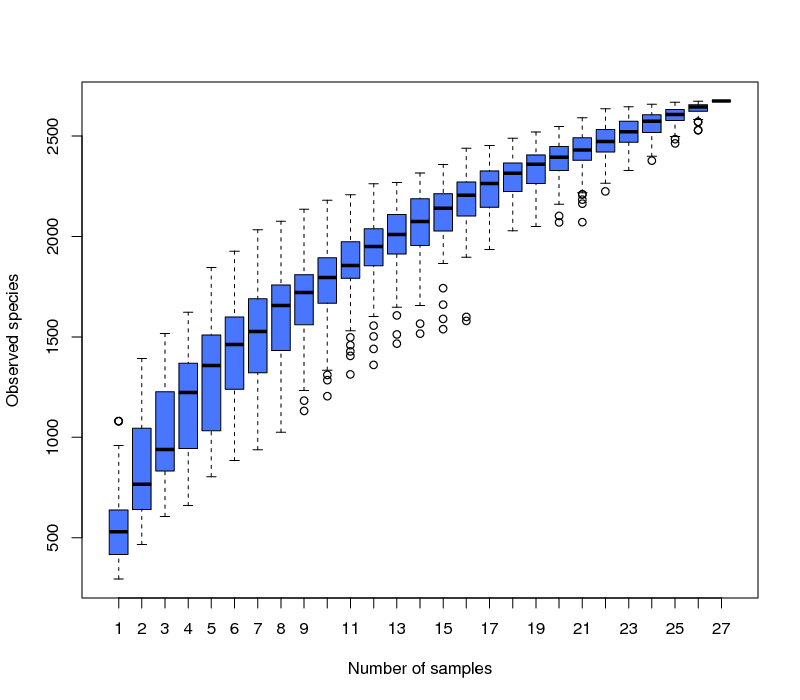

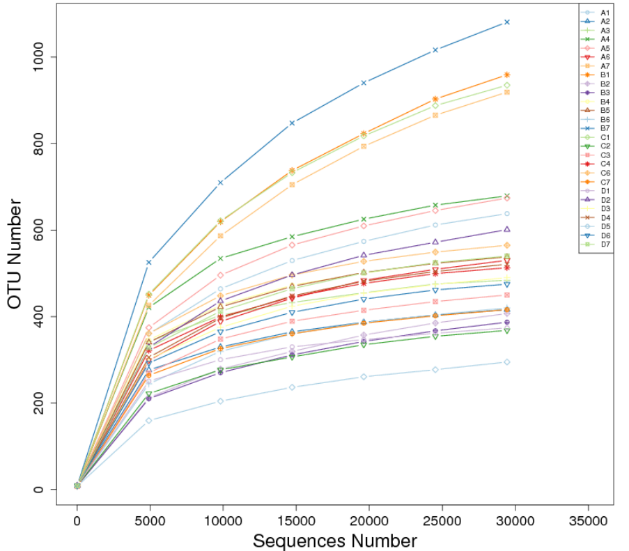
**

**A B**

**Figure S1**The species accumulation curve (A) and the rarefaction curve (B) of all samples.
